# Depression as the main manifestation of central pontine myelinolysis : A case report

**DOI:** 10.1192/j.eurpsy.2025.1684

**Published:** 2025-08-26

**Authors:** S. Walha, N. Halouani, S. Elloumi, S. Ellouze, M. Turki, J. Aloulou

**Affiliations:** 1 Psychiatry « B » department, Hedi Chaker University Hospital, Sfax, Tunisia

## Abstract

**Introduction:**

Centropontine myelinolysis (CPM) is an acute demyelinating neurological disorder that primarily affects the central bridge and is frequently associated with rapid correction of hyponatremia. Common clinical manifestations of CPM include spastic quadriparesis, dysarthria, pseudobulbar palsy and encephalopathy of varying degrees. In addition, CPM could be accompanied by neuropsychiatric manifestations, such as personality changes, thymic symptoms, acute psychosis, paranoia, hallucinations or catatonia, usually associated with additional brain damage, described as extrapontine myelinolysis (EPM).

**Objectives:**

Study the nature of comorbidity between CPM and mood disorders, particularly depression.

**Methods:**

We present the case of a patient hospitalized in the psychiatry department B of the Hedi Chaker hospital in Sfax in July 2024. He was been admitted at the request of a third party for behavioral disorders such as agitation and refusal of treatment.

**Results:**

This is Mr. S.BH aged 68, with a psychiatric family history of follow-up for unspecified psychiatric disorders in a niece. He has no psychiatric history, but has a somatic history, unmonitored high blood pressure as well as chronic constipation causing hyponatremia, which was quickly corrected 1 month ago. The latter was responsible for CPM objectified on the brain MRI requested by a free practice neurologist who consulted him for agitation. Our patient is married. He has been retired for a few months and previously worked as a farmer for 35 years. According to the family, the history of his illness dates back to March 2024, following professional stressors when he began to present multiple somatic complaints, with anorexia and weight loss as well as a tendency towards isolation. Since June 2024, following the CPM, he believed that the police wanted to harm him. As a result, he became anxious and agitated. At the interview: Slowed down on the psychomotor level, the contact was superficial, the mood was sad, his speech was provoked, poor and conveyed in a low voice verbalizing anhedonia, he presents congestive disorders and he refuses treatment and diet at the beginning. The patient obtained a score of 12 on < < the Geriatric Depression Scale GDS >> and a score of 12 on < <the mini-mental state examination MMSE >>.

**Conclusions:**

This case demonstrates that depression might represent the main manifestation of CPM, especially in the early stages of the disease, which should be taken into consideration when evaluating patients with acute abnormalities of sodium metabolism.

**Disclosure of Interest:**

None Declared

